# Spheroid growth in ovarian cancer alters transcriptome responses for stress pathways and epigenetic responses

**DOI:** 10.1371/journal.pone.0182930

**Published:** 2017-08-09

**Authors:** Trillitye Paullin, Chase Powell, Christopher Menzie, Robert Hill, Feng Cheng, Christopher J. Martyniuk, Sandy D. Westerheide

**Affiliations:** 1 Department of Cell Biology, Microbiology, and Molecular Biology, College of Arts and Sciences, University of South Florida, Tampa, Florida, United States of America; 2 Department of Pharmaceutical Sciences, College of Pharmacy, University of South Florida, Tampa, Florida, United States of America; 3 Department of Epidemiology and Biostatistics, College of Public Health, University of South Florida, Tampa, Florida, United States of America; 4 Center for Environmental and Human Toxicology & Department of Physiological Sciences, UF Genetics Institute, University of Florida, Gainesville, Florida, United States of America; University of Nebraska Medical Center, UNITED STATES

## Abstract

Ovarian cancer is the most lethal gynecological cancer, with over 200,000 women diagnosed each year and over half of those cases leading to death. These poor statistics are related to a lack of early symptoms and inadequate screening techniques. This results in the cancer going undetected until later stages when the tumor has metastasized through a process that requires the epithelial to mesenchymal transition (EMT). In lieu of traditional monolayer cell culture, EMT and cancer progression in general is best characterized through the use of 3D spheroid models. In this study, we examine gene expression changes through microarray analysis in spheroid versus monolayer ovarian cancer cells treated with TGFβ to induce EMT. Transcripts that included Coiled-Coil Domain Containing 80 (CCDC80), Solute Carrier Family 6 (Neutral Amino Acid Transporter), Member 15 (SLC6A15), Semaphorin 3E (SEMA3E) and PIF1 5'-To-3' DNA Helicase (PIF1) were downregulated more than 10-fold in the 3D cells while Inhibitor Of DNA Binding 2, HLH Protein (ID2), Regulator Of Cell Cycle (RGCC), Protease, Serine 35 (PRSS35), and Aldo-Keto Reductase Family 1, Member C1 (AKR1C1) were increased more than 50-fold. Interestingly, EMT factors, stress responses and epigenetic processes were significantly affected by 3D growth. The heat shock response and the oxidative stress response were also identified as transcriptome responses that showed significant changes upon 3D growth. Subnetwork enrichment analysis revealed that DNA integrity (e.g. DNA damage, genetic instability, nucleotide excision repair, and the DNA damage checkpoint pathway) were altered in the 3D spheroid model. In addition, two epigenetic processes, DNA methylation and histone acetylation, were increased with 3D growth. These findings support the hypothesis that three dimensional ovarian cell culturing is physiologically different from its monolayer counterpart.

## Introduction

Despite recent improvements in surgery and chemotherapy, ovarian cancer is still the leading cause of death from gynecological malignancy [1]. Due to poor detection methods and a lack of symptoms, most patients are diagnosed at advanced stages, when the tumor has metastasized and spread [2]. Studies suggest that in order for metastasis to occur, the cancer cells must undergo phenotypic changes modulated by the epithelial-mesenchymal transition (EMT) [3].

EMT is a distinct process whereby epithelial cells undergo changes in favor of mesenchymal properties [4]. This process is most commonly observed during developmental stages when epithelial cells must migrate and dedifferentiate, such as in the formation of the mesoderm during gastrulation [5]. In order to properly study this phenomenon, scientists have discovered multiple factors and signals which can induce EMT. Of these, the most popular inducer is transforming growth factor β (TGFβ) [6, 7]. The addition of TGFβ to epithelial cells induces transient EMT within hours of treatment through activation of the Smad pathway [8].

Although two dimensional (monolayer) tissue culture models are largely used to study the EMT process, evidence suggests that three dimensional (spheroid) culturing may be more physiologically relevant as it better emulates oxygen levels, pH conditions, glucose levels, extracellular matrix strength, and overall morphology of *in vivo* solid tumors [9–12]. This is especially the case when focusing on metastasis, tissue invasion, angiogenesis, and drug sensitivity [13–15].

At least a third of ovarian cancer patients present with ascites [16]. Ascites is the accumulation of fluid in the peritoneal cavity which may contain ovarian cancer cells, lymphocytes, and mesothelial cells in the form of single cells and aggregates [17]. Further studies revealed that ascites spheroids may cause secondary tumors due to their ability to adhere to extracellular matrix proteins via interaction between multiple integrins and their ligands [18, 19]. Here, we conducted a comprehensive gene expression analysis for the process of culturing HEY epithelial ovarian cancer cells in 3D vs. 2D cultures during the TGFβ-induced EMT process. Using subnetwork enrichment analysis, we identified stress pathways, DNA integrity pathways, and epigenetic processes as those most affected by 3D vs. 2D growth.

## Materials and methods

### Cells culture and treatments

The HEY human ovarian cancer cell line (kindly provided by Dr. Meera Nanjundan, University of South Florida, Tampa, FL) was authenticated by short tandem repeat (STR) DNA profiling (Genetica, Inc.) and was compared to ATCC and previously published profiles [20]. Cells were cultured in a humidified incubator containing 5% CO_2_ at 37°C in RPMI with 10% fetal bovine serum and 1% Pen-Strep-Glutamine. Spheroid formation was accomplished through the hanging drop method. Briefly, trypsinized HEY cells were resuspended at 1 x 10^6^ cells/mL in supplemented RPMI. Multiple 25 μl droplets of the cell solution were then placed onto plate lids, inverted, and incubated for 72 hours to allow cells to aggregate into spheroids. To induce EMT, a final concentration of 10 ng/μl TGFβ was added to cells and incubated for 72 hours as monolayer cell culture or hanging drops during the creation of spheroids. Following incubation, monolayer cells and spheroids were photographed using an Evos® FL Cell Imaging microscope (ThermoFisher Scientific) and then collected in 1X PBS.

### Quantitative RT-PCR

RNA was extracted from harvested cells using the RNeasy Mini Kit (Qiagen). RNA samples were then reversed transcribed with a High Capacity cDNA Reverse Transcription Kit (Applied Biosystems) per the manufacturer’s instructions. Subsequent samples were then diluted to 50 ng/ μL and used as a template for quantitative PCR (qPCR). qPCR was accomplished with a Step One Plus Real-time PCR system (Applied Biosystems) and SYBR® Green Supermix with ROX (BioRad) according to the manufacturer’s protocol. Relative mRNA levels were quantified for *ahnak2*, *akr1c1*, *ccdc80*, *hspa1a*, *hsph1*, *prss35*, *rgs2*, and *rrad* using gene-specific primers (S1 Table).

### Affymetrix GeneAtlas Platform and 3’IVT compatible U219 probe arrays

The oligonucleotide probe arrays used were the Affymetrix HG-U219 human array strips. These arrays consist of more than 530,000 probes detecting over 36,000 transcripts and variants-representing more than 20,000 genes.

### Sample processing and validation

HEY Human ovarian carcinoma cells treated with TGFβ were cultured as 2-dimensional monolayers or as 3-dimensional spheroids using the hanging drop method. Replicates of 4 separate experiments were performed on 4 different culturing days. Cells were then harvested and total RNA was isolated from all 8 samples using Qiagen’s RNeasy Mini Kit according to the manufacturer’s instructions. RNA integrity was verified with 1 μl of total RNA on RNA 6000 nanochips (Agilent) using The Agilent 2100 Bioanalyzer. All results for all 8 samples reported RNA Integrity Numbers (RIN) > 9. 100 ng of polyadenylated RNA was converted to cDNA and then amplified and labeled with biotin using Affymetrix 3’IVT Expression System according to the manufacturer’s instructions. Hybridization with the biotin-labeled RNA, staining, and scanning of the chips followed the procedure outlined in the Affymetrix technical manual. You can put this at the end of the methods on the micro array hybridization. All microarray data have been deposited into Gene Expression Omnibus (GSE80373).

### Data analysis

Scanned output files were visually inspected for hybridization artifacts and then annotated and normalized using Affymetrix Expression Console v1.3.1. Additionally, all QC metrics reported no outlier samples. Signal intensity was scaled to an average intensity of 500 during comparison analysis. Annotated expression data were assigned an ANOVA P value for the likelihood that any perceived difference was due to chance. The P values for all probe sets were exported to a text file and all pairwise comparisons of Bi-weight average signals were then aligned in MS Excel. For the comprehensive analysis, P < 0.05 was identified as having a linear change (increased or decreased) for the comparison. This analysis reported ~3,329 genes that showed differential regulation greater or less than 2-fold. To increase stringency, only genes whose fold-change was greater than or less than four (p < 0.05) were considered to be differentially expressed, reducing the pool to 493 candidate genes.

Data were analyzed using different visualization techniques that included hierarchical clustering, principle component analysis (PCA), and volcano plots. All analyses were conducted in JMP Genomics v7.0. Transcripts that showed p<0.01 were used in the two-way clustering using the Fast ward algorithm after each row was centered to a mean of zero (0) and variance scaled to one. Results from hierarchical cluster analyses were visualized using heat map dendrograms, and biological replicates were partitioned into groups based on similarity (i.e., individuals clustered together are most similar). PCA used spatially weighted averages and were conducted on normalized expression values, the same dataset used in the cluster analysis. Volcano plots were generated in JMP Genomics 7.0 following an ANOVA to identify differentially expressed transcripts.

### Pathway Studio analysis

Pathway Studio 9.0 (Elsevier) and ResNet 10.0 were used for sub-network enrichment analysis (SNEA) of cell processes [21]. The option of “best p value, highest magnitude fold change” in Pathway Studio was used for duplicated probes. Transcripts were successfully mapped using GeneBank ID. SNEA was performed to identify gene networks that were significantly different. A Kolmogorov–Smirnov test with 1000 permutations was conducted to determine whether certain networks were preferentially regulated compared to the background reference probability distribution. Networks were constructed based on common regulators of expression and regulators of specific cell processes. The enrichment P-value for a gene seed was set at P < 0.05. Additional details on the use of SNEA can be found in Langlois and Martyniuk [22].

## Results and discussion

### HEY cells treated with TGFβ have distinct gene expression profiles when grown as 3D spheroids vs. 2D monolayers

**Fig 1:**
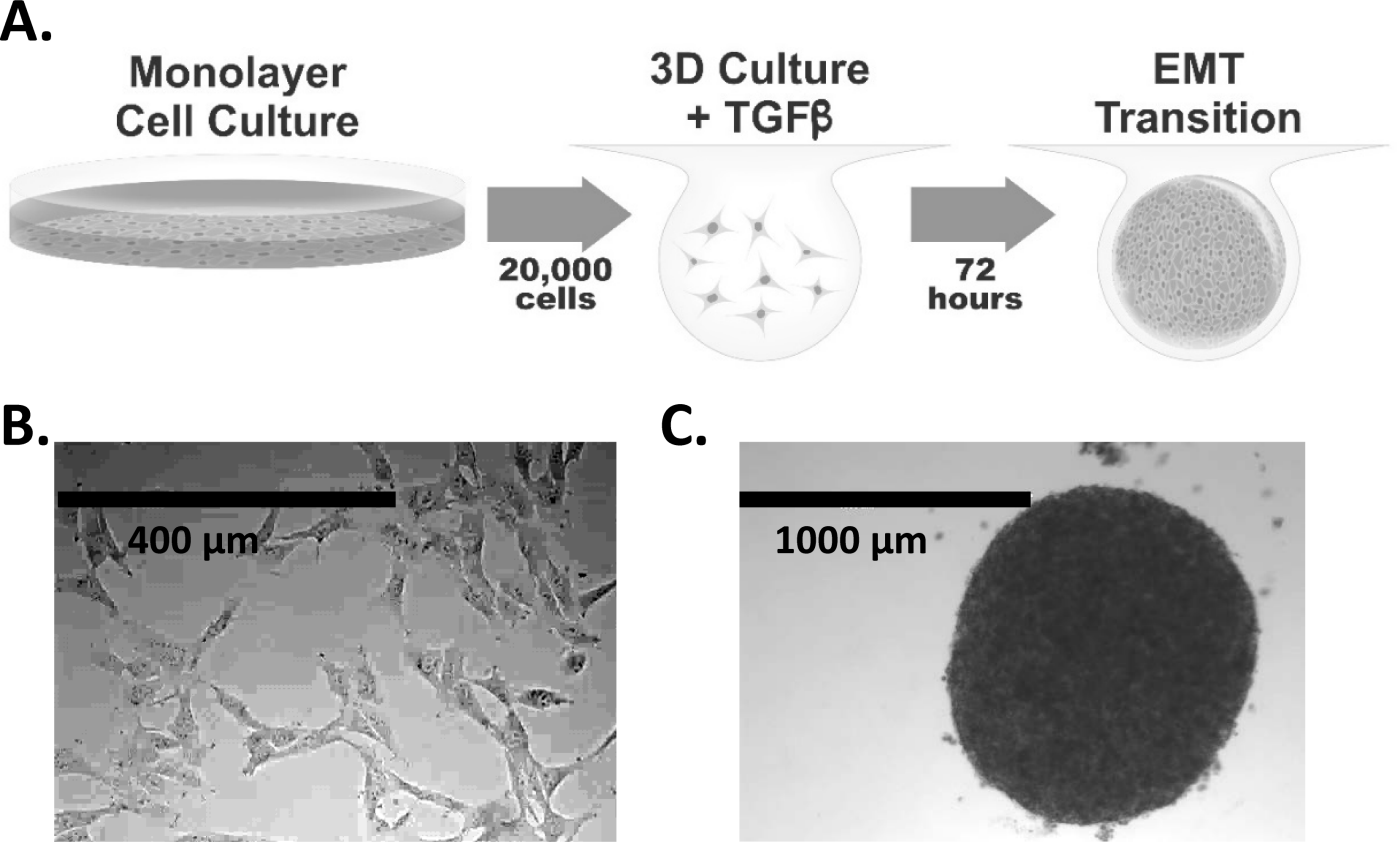
Establishment of three-dimensional mesenchymal cell populations from confluent monolayers. A) HEY cells grown in a monolayer are released and then suspended in drops of culture media containing TGFβ (20,000 cells per drop) using the hanging drop method. After 72 hours, the resulting 3D spheroids are then assayed. B) Image of HEY cells grown in monolayer culture. C) Image of HEY cells grown as a 3D spheroid and treated with TGFβ.

**Fig 2:**
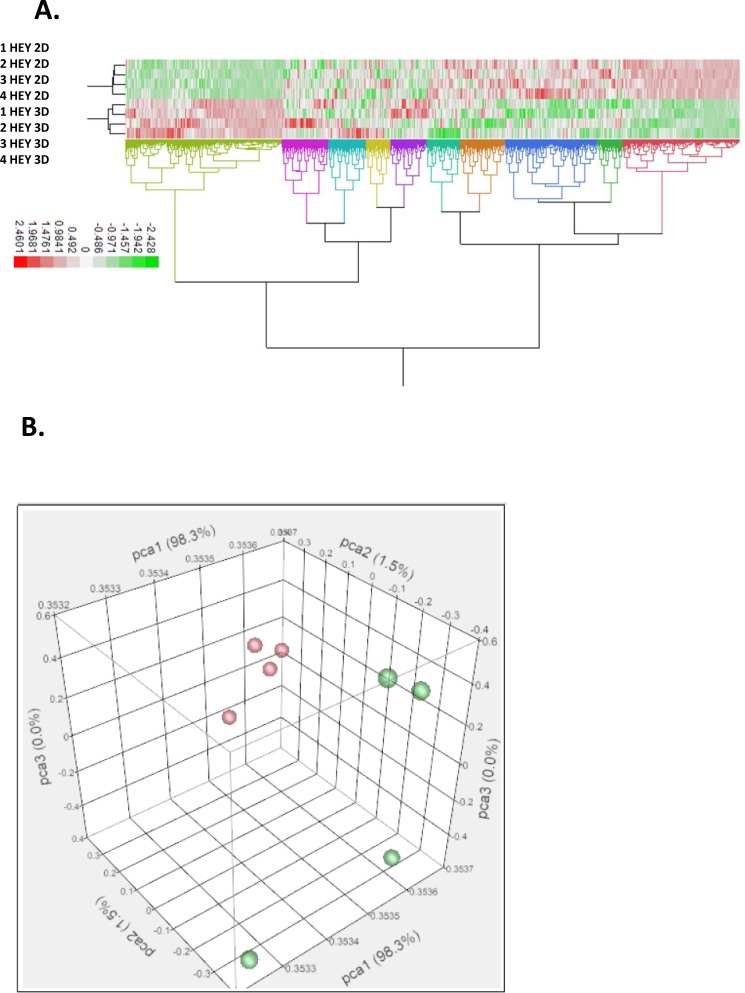
HEY cells treated with TGFβ have distinct gene expression profiles when grown as 3D spheroids vs. 2D monolayers. A) Hierarchical clustering of expression profiles. Clustering revealed that each of the two groups (4 biological replicates per group) form into distinct clades based on expression. B) Principle component analysis for expression profiles. Variability in transcriptome response separates strongly along the first PCA1. Red color is HEY 3D biological replicates and green color is HEY 2D biological replicates. The four biological replicates for HEY 2D are more closely related in expression as compared to HEY 3D.

To examine whether growth of HEY ovarian cancer cells as 3D spheroids vs. 2D monolayers could influence gene expression, we performed microarray analysis on four biological replicates of cells grown under each condition. To create spheroids, the hanging drop method [23] was used as outlined (Fig 1). We find that HEY cells treated with TGFβ have distinct gene expression profiles, depending on whether they are cultured under 2D vs. 3D conditions (Fig 2). Hierarchical clustering reveals that each of the two groups form into distinct clades based on gene expression (Fig 2A). Additionally, principle component analysis shows that biological quadruplicates for HEY 2D and HEY 3D samples are more similar to themselves than to each other (Fig 2B). Volcano plot analysis shows that there are numerous genes between the two groups that have a high fold change and that are also statistically significant (S1 Fig).

**Fig 3:**
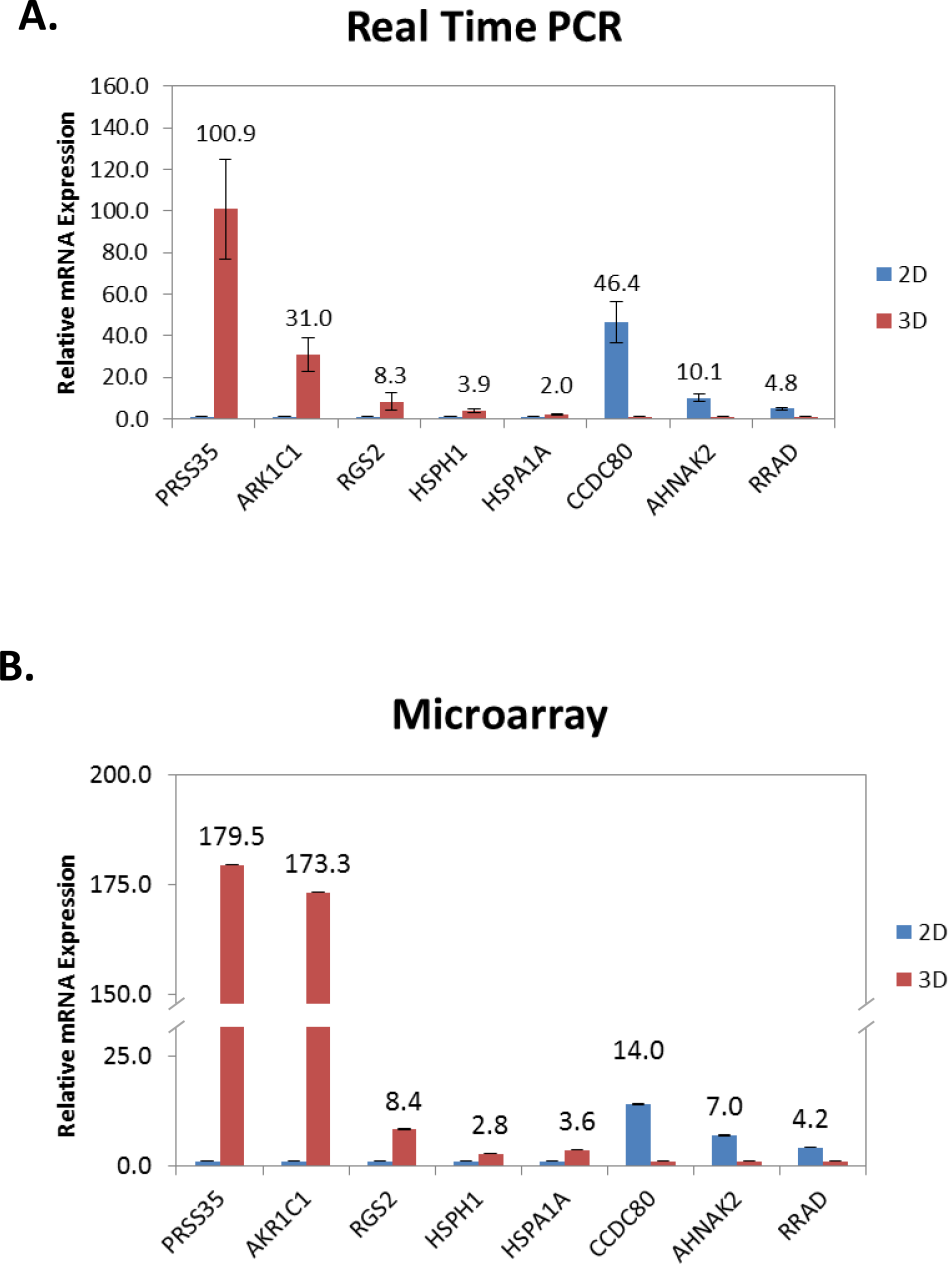
Real time PCR analysis validates microarray results. A) Real time PCR results for select genes that are moderately to highly affected by 3D vs. 2D growth. B) Microarray results are plotted for the same genes.

RT-qPCR was performed on eight different genes which were found to be up or downregulated by varying degrees upon 3D culturing to validate our microarray analysis (Fig 3). The eight genes we chose to validate include two highly upregulated genes, the serine protease PRSS35 and the aldo-keto reductase AKR1C1, as well as the highly downregulated E-cadherin regulator CCDC80. Additionally, we chose some genes that were more moderately regulated, including the chaperones HSP110 and HSP70, the G-protein signaling inhibitor RGS2, the fibroblast growth factor secretion regulator AHNAK2 and the apoptotic inducer RRAD. Our results show that similar trends are observed via RT-qPCR for these genes as compared to our microarray results. Overall, our data show that HEY ovarian cancer cells, treated with TGFβ to induce EMT, show dramatic differences in gene expression profiles when grown as 3D spheroids vs. standard 2D culture.

### Transcripts affected by 3D culturing may enhance the tumorigenicity of HEY cells

Growth as 3D spheroids leads to both the upregulation and downregulation of gene expression (Table 1. For a complete list of gene expression data see S2 Table). The most highly upregulated gene under 3D growth conditions in HEY cells is AKR1C1 (Aldo-Keto Reductase Family 1, Member C1), part of a family of cytosolic NADP(H)-dependent oxidoreductases that is involved in detoxification of xenobiotics, steroids, and polycyclic aromatic hydrocarbons [24]. Increased expression of AKR1C1 is associated with the development of cisplatin resistance in human ovarian carcinoma cells [25]. Previous work shows that ovarian cancer cells, grown in 3D, are more resistant to chemotherapy treatment [26]. It is therefore plausible that the increased expression of AKR1C1 upon ovarian cancer cell 3D growth may promote chemotherapy resistance. PRSS35 (Protease, Serine 35), another highly upregulated gene upon 3D growth, belongs to the trypsin class of serine proteases. This protease is highly expressed in the mouse ovary [27]. Proteases play an important role in proteolysis that is essential for tissue remodeling that occurs during EMT by breaking down the extracellular matrix. While metalloproteases play the largest role in this process, serine proteases also contribute [28]. We postulate that enhanced expression of PRSS35 under 3D growth conditions may promote ovarian EMT through the proteolytic digestion of cell attachments. A transcript that was highly downregulated upon 3D growth is CCDC80 (Coiled-Coil Domain Containing 80). Interestingly, CCDC80 null mice develop thyroid adenomas and ovarian carcinomas, and CCDC80 gene expression is decreased in human ovarian cancer samples as compared to normal ovarian samples [29]. The mechanism for the tumor suppressor properties of CCDC80 may be through its ability to induce E-cadherin expression [29]. E-cadherin provides crucial cell-cell adhesion to hold epithelial cells tightly together, and enhanced E-cadherin expression promotes the epithelial phenotype over the cancer-promoting mesenchymal phenotype [30]. Collectively, we propose that regulation of these and other genes upon 3D growth may enhance the tumorigenic properties of ovarian cancer cells.

**Table 1:** The most differentially expressed transcripts in the HEY 3D compared to HEY 2D.

*Direction of Change*	*Gene*	*Role*	*Fold Change*	*p-value*
Down-regulated	CCDC80	Heparin binding and fibronectin binding	-17.39	2.87E-09
	SLC6A15	Amino acids transport	-14.75	2.3E-08
	SEMA3E	Axon guidance ligands	-14.67	9.33E-09
	TENM2	Protein homodimerization activity	-13.71	7.28E-10
	PLCB4	Catalyzes the formation of inositol 1,4,5-trisphosphate and diacylglycerol	-13.13	2.43E-08
	MIR17	Involved in cancer and cell carcinomas	-13.09	2.91E-08
	PIF1	5' to 3' DNA helicase	-13.02	1.09E-08
	IL1RAP	Associates with interleukin to mediate NF-κB signaling	-11.49	1.16E-08
	GPR126	G-protein orphan receptor	-10.83	6.66E-09
	FBN2	Component of connective tissue microfibrils	-10.45	2.36E-08
	MIR1304	mRNA regulation	-9.92	1.22E-08
	TRMT13	Methyltransferase activity	-9.22	7.77E-09
	DLEU2	Associated with chronic lymphocytic leukemia	-9.09	4.32E-09
	TIGD1	Unknown	-8.98	6.51E-08
	TPM1	Involved in the contractile system of striated and smooth muscles	-8.91	9.32E-08
	KRTAP2	Formation of a rigid and resistant hair shaft	-8.78	4.61E-09
	RIMS2	Ion channel binding and Rab GTPase binding	-8.77	6.88E-10
	ATP8B1	Transport of phosphatidylserine and phosphatidylethanolamine	-8.60	7.27E-10
	AHNAK2	Cell differentiation	-8.56	8.9E-09
	AKAP12	Scaffold protein in signal transduction	-8.08	5.87E-09
Up-regulated	MRPS6	Protein synthesis in mitochondria	18.96	5.7E-10
	TNFSF15	Cytokine inducible by TNF and IL-1α	20.18	2.85E-08
	RANBP3L	Binding protein	20.29	3.27E-09
	SLC1A3	Termination of excitatory neurotransmission in central nervous system	22.47	1.07E-09
	TMEM171	Unknown	24.17	7.76E-10
	AKR1B1	Catalyzes the reduction of aldehydes	24.76	4.53E-10
	F2RL1	Receptor for trypsin and trypsin-like enzymes coupled to G proteins	25.68	5.85E-10
	S100A4	Cell cycle progression and differentiation	28.15	5.7E-10
	PTGS2	Prostaglandin biosynthesis	29.55	3.06E-08
	SOD2	Converts superoxide byproducts to H2O2	32.39	4.72E-09
	CXCR4	CXC chemokine receptor	35.08	6.24E-10
	IL8	Neutrophil chemotactic factor	55.81	7.76E-10
	SPOCK1	Protease inhibition	56.98	6.76E-10
	NADK2	Catalyzes the phosphorylation of NAD to yield NADP	57.98	2.16E-08
	GLS	Catalyzes the hydrolysis of glutamine to glutamate	59.61	6.06E-08
	ITGB8	Mediate cell-cell and cell-extracellular matrix interactions	60.23	2.1E-09
	ID2	Cellular growth, senescence, differentiation, apoptosis, angiogenesis	70.77	4.63E-09
	RGCC	Regulates cell cycle, induced by p53	78.93	5.85E-10
	PRSS35	Protease activity	179.46	5.85E-10
	AKR1C1	Conversion of aldehydes and ketones to their corresponding alcohols	198.09	5.7E-10

Transcripts are organized as those down-regulated and up-regulated between the two groups. The gene, as well as the role of the protein in the cell is provided in addition to the relative fold change and p-value. For a complete list of gene expression changes see S2 Table.

### Gene-set enrichment analysis reveals novel pathways that are enriched upon HEY cell growth as 3D spheroids

Gene set enrichment analysis suggested that pathways downregulated by more than 15% include branched chain amino acid metabolism, folate biosynthesis, fatty acid oxidation, and the mevalonate pathway, while pathways upregulated in the 3D cells include those related to tumor necrosis factor receptor (TNFR) superfamily member 1A and 6 (for a complete list of gene set enrichment pathways identified see S3 Table). These pathways were increased more than 20% with 3D growth in HEY cells, and they have all been related to cancer growth. Branched chain amino acids, such as leucine, positively regulate the mammalian-target-of-rapamycin (mTOR) pathway [31]. The mTOR pathway is involved in regulating cellular functions involved in growth and proliferation, and upregulation of this pathway is commonly observed in human cancers [32]. Folate plays a role in nucleotide synthesis and methylation, making it essential to rapidly growing cancer cells [33]. Fatty acid oxidation is required for functional angiogenesis and is utilized by cancer cells to overcome metabolic stress to proliferate [34]. The mevalonate pathway is responsible for converting acetyl-coenzyme A into isoprenoids, which are required for cholesterol and steroid synthesis [35]. LDL-cholesterol accumulates in cancer tissues and is associated with migration, proliferation, and loss of adhesion from the primary tumor which are vital steps in the epithelial to mesenchymal transition [35]. Upregulation of TNFR family members allows signaling via the NF-κB transcription factor, which can promote tumorigenesis through the activation of the expression of genes involved in processes including cell proliferation, migration and anti-apoptosis [36]. Therefore, we conclude that multiple cellular pathways that are altered upon 3D growth could increase tumorigenicity.

### EMT genes are altered upon spheroid culturing in ovarian cancer

**Fig 4:**
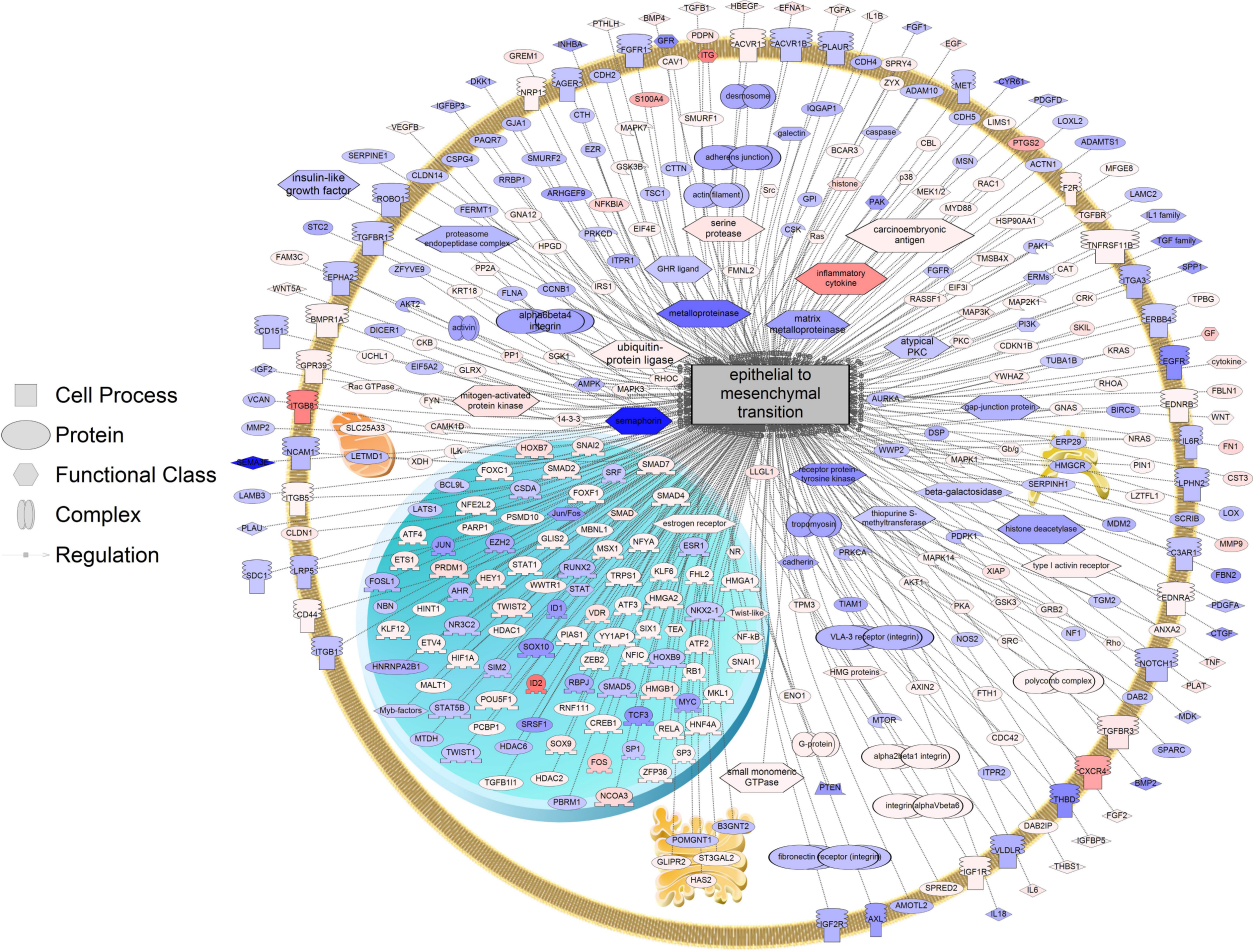
Expression of genes related to EMT in 3D vs. 2D growth. All these genes in the pathway were differentially expressed at P<0.001 in 3D and are related to the process of EMT. Red indicates the gene is up-regulated and blue indicates the gene is down-regulated. All genes for pathways are listed in S4 Table.

Unique sub-networks underlie the transition from 2D to 3D growth in HEY cells (Table 2, for a complete list of enriched cell networks see S3 Table). We note that many of the genes involved in EMT are differentially expressed upon formation of spheroids as compared to growth in monolayer (Fig 4). EMT transcription factors downregulate the expression of cell-cell adhesion proteins and upregulate metastatic proteins, leading to cancer progression [37]. Importantly, a number of EMT transcription factors are enhanced upon growth in 3D, including Snail 1 (SNAI1), Snail 2 (SNAI2), ZEB2, TCF3, and SIX1 (S4 Table). Supporting this finding, in recently published work, we show via qPCR that EMT transcription factors, while slightly upregulated upon TGFβ treatment under 2D growth conditions, are upregulated more significantly when grown in 3D [38]. Thus, 3D growth, through enhanced expression of EMT transcription factors, is likely to allow for a stronger induction of EMT by TGFβ.

**Table 2:** Examples of cell processes differentially expressed between HEY 2D and HEY 3D cells.

*Theme*	*Gene Set Seed*	*Total # of Neighbors*	*# of Measured Neighbors*	*Median change*	*p-value*
Autophagy	autophagy	418	402	1.08	5.9E-06
	autophagic cell death	78	75	1.15	0.00076
Cancer and Aging	senescence	651	596	1.05	7.79E-08
	cell aging	251	225	1.06	2.1E-07
	oncogenesis	523	486	1.05	1.36E-05
	epithelial to mesenchymal transition	551	522	-1.0087	1.99E-07
Cell Cycle	S phase	857	797	1.05	4.38E-09
	interphase	196	192	-1.05	1.13E-08
	G1/S transition	611	558	1.07	1.73E-06
	G2 phase	171	164	1.07	2.51E-05
	cell cycle checkpoint	161	150	1.06	4.1E-05
	exit from mitosis	62	59	1.06	0.000284
Chromosome Separation	spindle assembly	507	478	-1.06	1.14E-11
	centriole duplication	106	103	-1.10	6.98E-07
	mitotic spindle assembly	59	58	-1.18	2.98E-05
	microtubule/kinetochore interaction	19	19	-1.54	0.000872
DNA Damage and Repair	response to DNA damage	460	428	1.07	1.32E-12
	DNA repair	654	609	1.05	1.3E-10
	genome instability	292	272	1.06	4.09E-08
	genetic instability	113	111	1.08	3.76E-07
	nucleotide-excision repair	120	113	1.09	7.58E-06
	DNA damage checkpoint	126	122	1.06	6.2E-05
Protein Modification	protein degradation	470	449	1.06	7E-08
	ubiquitin-dependent protein degradation	193	186	1.16	1.23E-05
	protein ubiquitination	89	88	1.15	3.27E-05
	protein sumoylation	167	158	1.13	4.71E-05
Stress	response to oxidative stress	166	159	1.06	3.55E-05
	heat-shock response	65	59	1.14	0.000722
Transcription	poly(A)+ mRNA-nucleus export	84	77	-1.13	9.86E-05
	Polymerase II transcription	75	70	1.13	0.000393
Other	adipocyte differentiation	394	367	1.06	5.11E-06
	wall integrity	51	51	1.37	5.74E-06
	myoblast differentiation	212	196	1.08	1.85E-05
	nucleocytoplasmic transport	122	112	1.10	2E-05
	adherens junction assembly	52	50	1.12	2.79E-05
	skin development	69	67	1.10	0.000175
	blood vessel barrier	28	28	1.26	0.000545
	leiomyocyte adhesion	25	24	-1.14	0.000756

Provided are the total number of neighbors within a network, number of neighbors measured on the microarray, the median fold change of the network, and the p-value. All cell processes differentially expressed between cells are provided in S3 Table. Major themes included those related to cell cycle, DNA damage and repair, and stress.

### Transcriptional networks involving stress pathways are altered in HEY cell growth as 3D spheroids

**Fig 5:**
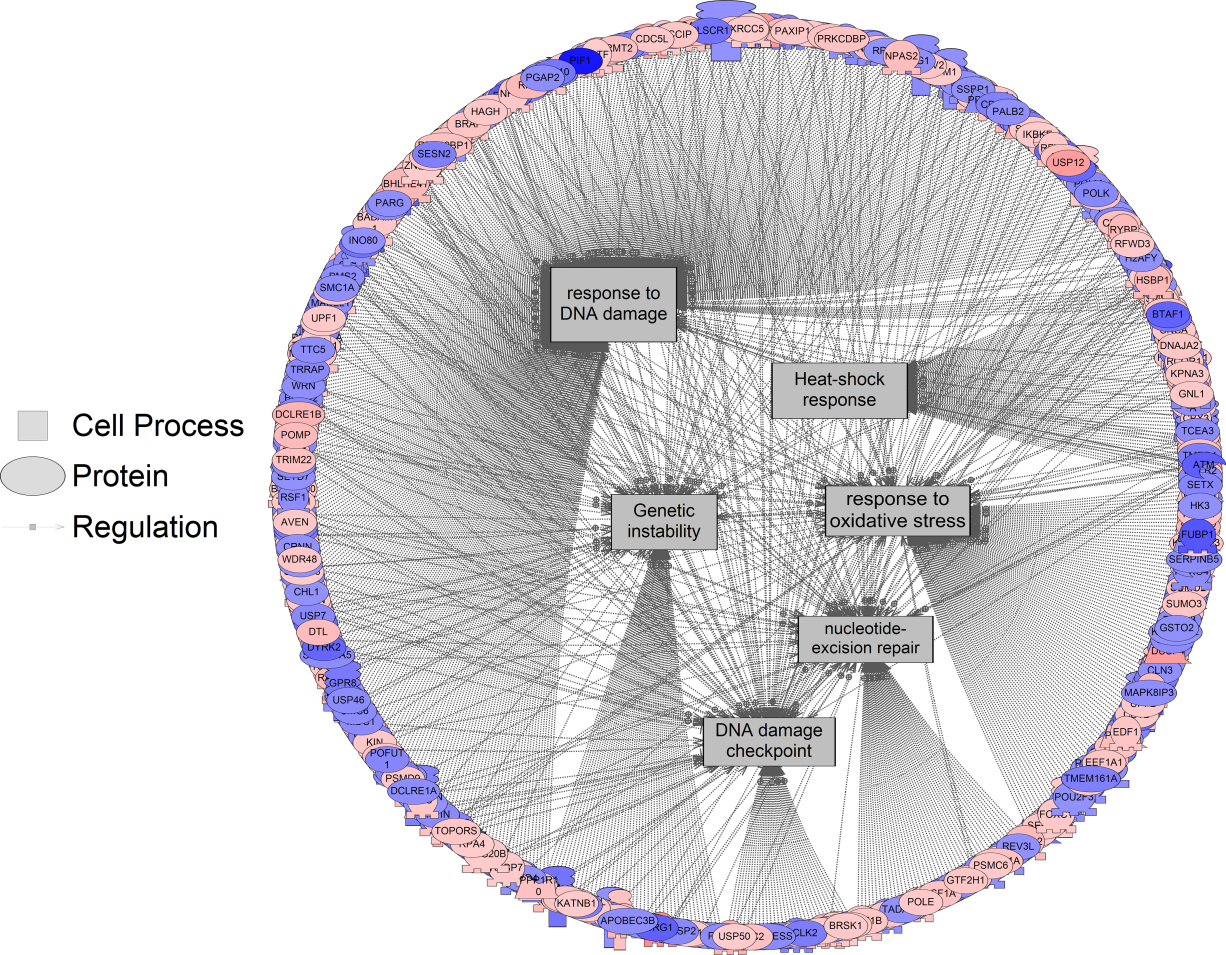
Gene networks related to stress are significantly enriched following sub-network enrichment analysis when comparing 3D vs. 2D growth. Red indicates the gene is upregulated and blue indicates the gene is downregulated. All genes for the pathways are listed in S4 Table.

Interestingly, a number of the identified sub-networks are related to the response to stress (Fig 5, for a complete list of gene networks related to stress see S4 Table). The finding that stress responses are activated upon 3D growth is not surprising, given that growth under these conditions is likely to cause nutrient limitation and hypoxia, among other cellular stresses.

Transcripts in the network that are related to the oxidative stress response include peroxiredoxin 2 (PRDX2) (Fold change = +2.2), catalase (CAT) (+1.3), superoxide dismutase 1, soluble (SOD1) (+1.2), and glutathione S-transferase omega 1 (GST01) (-1.3). Transformation may result from the exposure of cells to reactive oxygen species [39]. Additionally, it has been hypothesized that the rise in cancer diagnosis for older individuals may be due to a lifetime of DNA damage by reactive species accumulated endogenously and exogenously [40]. Oxidative stress activates ERK/MEK and PI3K/AKT pathways and impacts signaling proteins important in cancer such as Ras, Raf, p53, PKC, c-Myc, and Nrf2 [41–43]. The changes detected in the oxidative stress pathway upon growth of HEY cells in 3D may thus promote tumorigenic properties of the cells.

Also changed in 3D cells are transcripts that are associated with the heat shock response. Some examples include heat shock protein 90kDa alpha (cytosolic), class A member 1 (HSP90AA1) (+1.16), heat shock 27kDa protein 1(HSPB1) (+1.20), and heat shock transcription factor 1 (HSF1) (+1.28). Recent studies have suggested that many cancer types utilize activation of heat shock factor 1 (HSF1), the master regulator of the heat shock response, to stabilize oncogenes within the cell [44]. The heat shock response is a highly conserved response to specific environmental stressors such as heat shock, heavy metals, and oxidative stress [45]. HSF1 promotes the transcription of heat shock protein genes, many of which encode molecular chaperones. Increased chaperone expression provides a cytoprotective response in advanced cancers with acidotic, hypoxic, and nutrient-deprived microenvironments [46]. Elevated levels of one or more chaperones are commonly found in both solid cancer tumors and hematological malignancies [47–51]. In fact, overexpression of HSP27, HSP70, or HSP90 correlates with poor prognosis and may contribute to drug resistance in some cancer types [52–55]. Activation of the heat shock response in spheroids may promote cell survival, in the face of multiple cellular stresses, through enhanced expression expression of HSF1 target genes, including molecular chaperones. Further analysis of this spheroid model could offer valuable insight into how HSF1 and HSPs could be utilized as targets for chemotherapy in ovarian cancer patients.

Other examples of stress-related genes that show a high magnitude of response in terms of downregulation include the PIF1 5'-to-3' DNA helicase homolog (*S*. *cerevisiae*) (-13.0), epidermal growth factor receptor (-4.6), far upstream element (FUSE) binding protein (-4.4), and cysteine-rich, angiogenic inducer (-4.4). Increasing transcripts in the network included nuclear factor of kappa light polypeptide gene enhancer in B-cells inhibitor, alpha (+10.3), FBJ murine osteosarcoma viral oncogene homolog (+14.7), prostaglandin-endoperoxide synthase 2 (prostaglandin G/H synthase and cyclooxygenase) (+29.5), and interleukin 8 (+49.8). Many of these aforementioned transcripts have been shown to play critical roles in the progression of cancer. For example, PIF1 is a DNA-dependent adenosine triphosphate (ATP)-metabolizing enzyme that is required for proper replication and repair during cell division. Studies show that this protein inhibits S-phase progression and reduces proliferation rates of RAS oncogene-transformed fibroblasts [56], and may therefore be a novel drug target for cancer therapy. Here, the downregulation of PIF1 mRNA may lead to increased proliferative activity during the transition from a non-cancerous cell to a cancerous cell. Our network analysis also identifies DNA duplex unwinding (down regulated -1.49) and S-M checkpoint (down-regulated -1.59) as processes significantly down-regulated in 3D spheroids, processes which may be related to changes in PIF1 expression. In terms of ovarian cancer, there are many studies implicating epidermal growth factor receptor (EGFR) as a key regulator of cell differentiation Specifically, EGF-induced EMT increases phosphorylation of Akt, ERK1/2, and S6 ribosomal protein, which alters ovarian cancer cell proliferation and differentiation [57]. Additionally, elevated EGFR expression in tumor stroma is linked to aggressive epithelial ovarian cancer in patients and relates to Ki-67 expression in tumor cells [58]. In terms of up-regulated transcripts, notable transcripts included FBJ murine osteosarcoma viral oncogene homolog, a member of the Fos family of transcription factors. In cancer, this gene is a proto-oncogene implicated in cell proliferation and transformation [59]. Lastly of interest in the network was IL8, a gene that showed a dramatic increase in expression of ~50-fold in 3D cells. Polymorphisms in this gene have been shown to be associated with a significantly higher risk of ovarian cancer [60, 61]. Studies also show that increases in IL8 is associated with increased tumor growth and metastases [62].

Various responses related to DNA integrity were altered, including the response to DNA damage, genetic instability, nucleotide excision repair, and the DNA damage checkpoint pathway. The hypoxic environment created within spheroids may lead to increased DNA damage. Spheroid culturing has shown to alter chromatin packaging which in turn improves DNA repair through what is known as the cell “contact” effect [63]. DNA damage via metabolic products and by-products, such as ROS, may decrease replication fidelity, resulting in increased mutagenesis. Mutations and chromosomal abnormalities can increase the risk of cancer through the activation of oncogenes or the inactivation of tumor suppressor genes. Complex DNA-damage response mechanisms have evolved in order to isolate and repair these mutations. One such mechanism is the nucleotide excision repair (NER) pathway. NER is able to eradicate a variety of DNA lesions due to its ability to circumvent recognition of the lesion itself and focus on a set of commonalities shared by many different lesions [40]. Our analysis showed a significant enrichment of the NER pathway when cells are cultured as 3D spheroids.

### Sub-network enrichment analysis identifies that genes involved in epigenetic processes are enriched upon HEY cell growth as 3D spheroids

**Fig 6:**
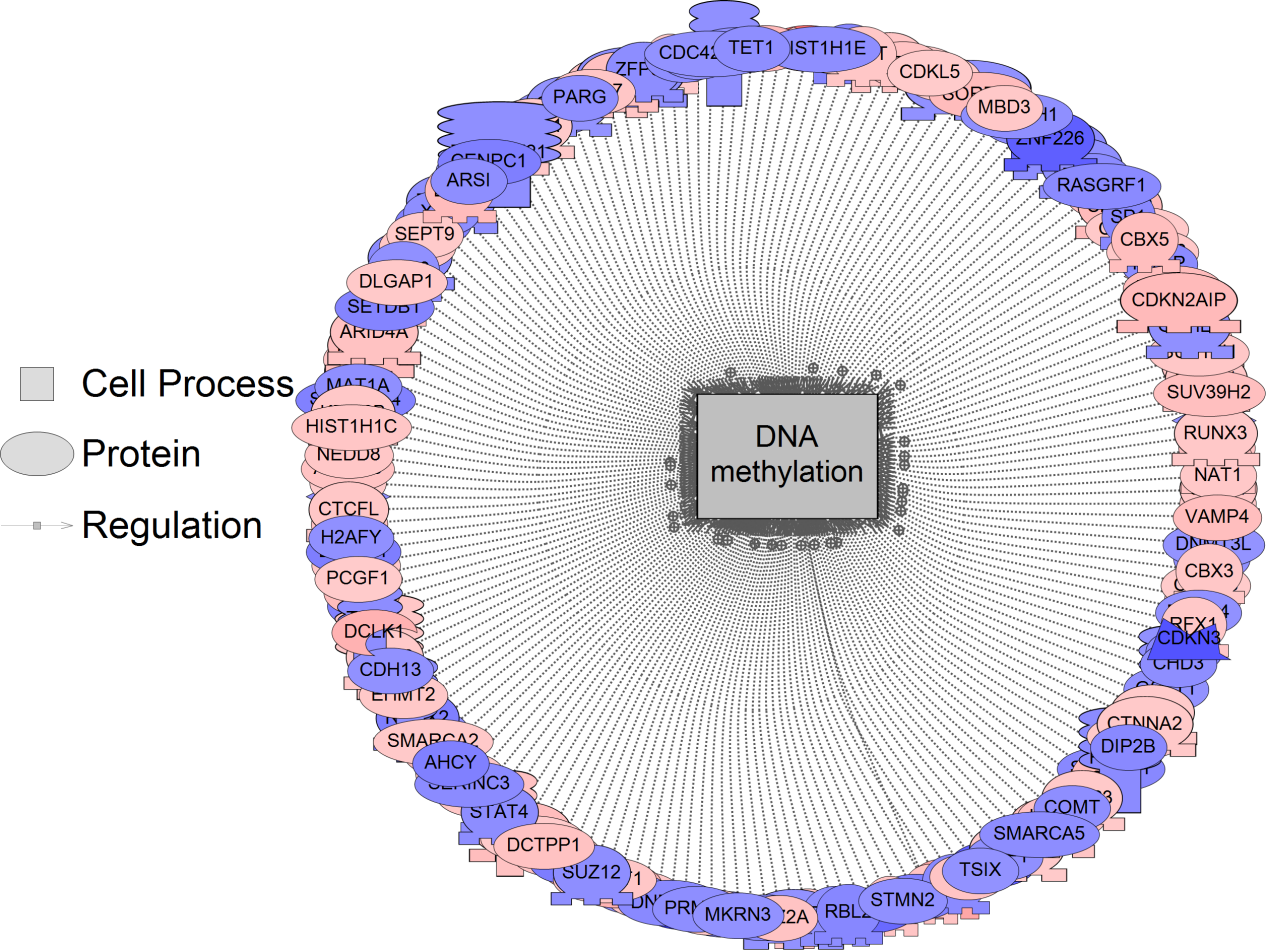
DNA methylation was significantly enriched following sub-network enrichment analysis when comparing 3D vs. 2D growth. DNA methylation was preferentially increased approximately 4% (197 genes measured, P = 0.006) in the 3D group at the level of the transcriptome based on the sub-network enrichment analysis. Red indicates the gene is up-regulated and blue indicates the gene is down-regulated. All genes for DNA methylation are listed S4 Table.

Epigenetic alterations can play a key role in promoting transformation and tumor growth [64–66], although the underlying mechanisms are still being worked out. We find two epigenetic processes, DNA methylation and histone acetylation, to be enriched upon 3D growth. A number of genes are both up- and downregulated related to DNA methylation, and this process is affected by about 4–5% (Fig 6, for a complete list of all genes in the DNA methylation pathway see S4 Table). DNA methylation changes are seen in several cancer types and have been linked to changes in gene expression in highly metastatic tumors [67].

**Fig 7:**
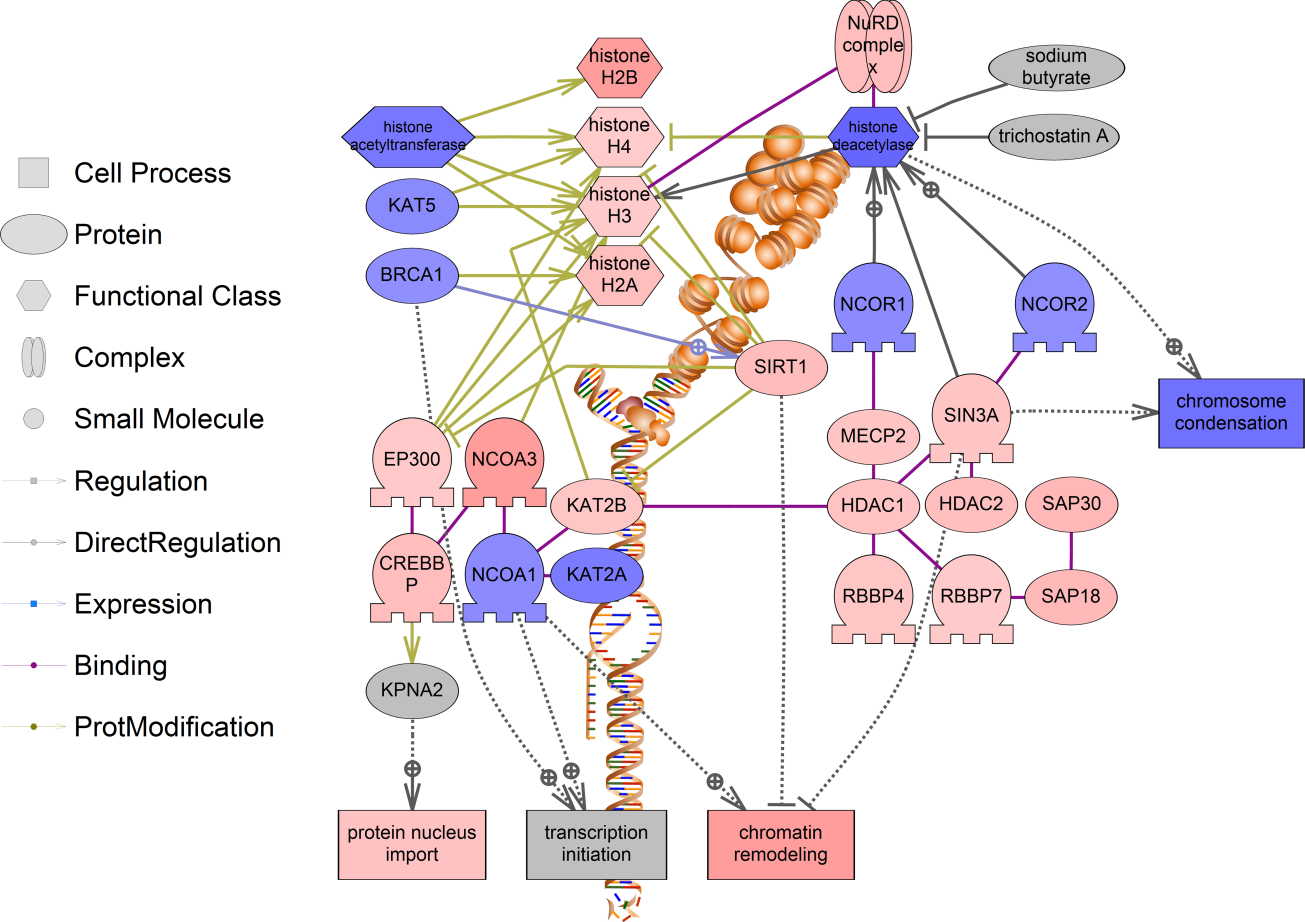
Pathway for histone acetylation. A number of histone modifying enzymes were increased in expression in the 3D group, and this may be reflective of chromatin remodeling during cancer progression. Red indicates the gene or process is up-regulated and blue indicates the gene or process is down-regulated. All genes for histone acetylation pathways are listed in S4 Table.

The process of histone acetylation is also significantly affected in both positive and negative fashions (Fig 7, for a complete list of all genes in the histone acetylation pathway see S4 Table). Histone acetylation has important roles in diverse processes including gene regulation, DNA damage repair, and DNA replication. Significant correlations between cancer patient survival and histone acetylation have been shown in several studies, although it has been associated with both better and worse prognoses depending on the specific cancer type [68, 69]. Thus, we conclude that growth as 3D spheroids may affect the epigenetic status of cancer cells, which could alter the tumorigenic properties of the cells.

## Conclusions

Spheroid formation more closely mimics that of an *in vivo* tumor due to the cells ability to form an extracellular matrix and cell adhesions. Here, using the HEY ovarian cancer cell line, we show that 3D growth affects a number of cellular processes, including the EMT process, multiple cellular stress pathways, DNA integrity pathways, and epigenetic pathways. As these pathways all could affect tumorigenesis and the response to chemotherapies, our studies suggest that using 3D culture instead of 2D monolayers may be more informative in studying the properties of ovarian cancer cell lines.

## Supporting information

S1 Fig Volcano plot analysis of HEY 2D vs. HEY 3D. (TIF)

S1 Table Primers for qPCR. (DOC)

S2 Table Complete list of transcripts affected by 3D culturing. (XLSX)

S3 Table Complete lists of geneset enrichment pathways and subnetwork enrichment analyses. (XLSX)

S4 Table Complete list of sub-networks that are related to the response to stress, the DNA methylation pathway and the histone acetylation pathway. (XLSX)
