# Supplementary figures and images for: Spheroid growth in ovarian cancer alters transcriptome responses for stress pathways and epigenetic responses
Source: PLoS One. 2017 Aug 9;12(8):e0182930. doi: 10.1371/journal.pone.0182930 (PMC5549971; doi:10.1371/journal.pone.0182930)

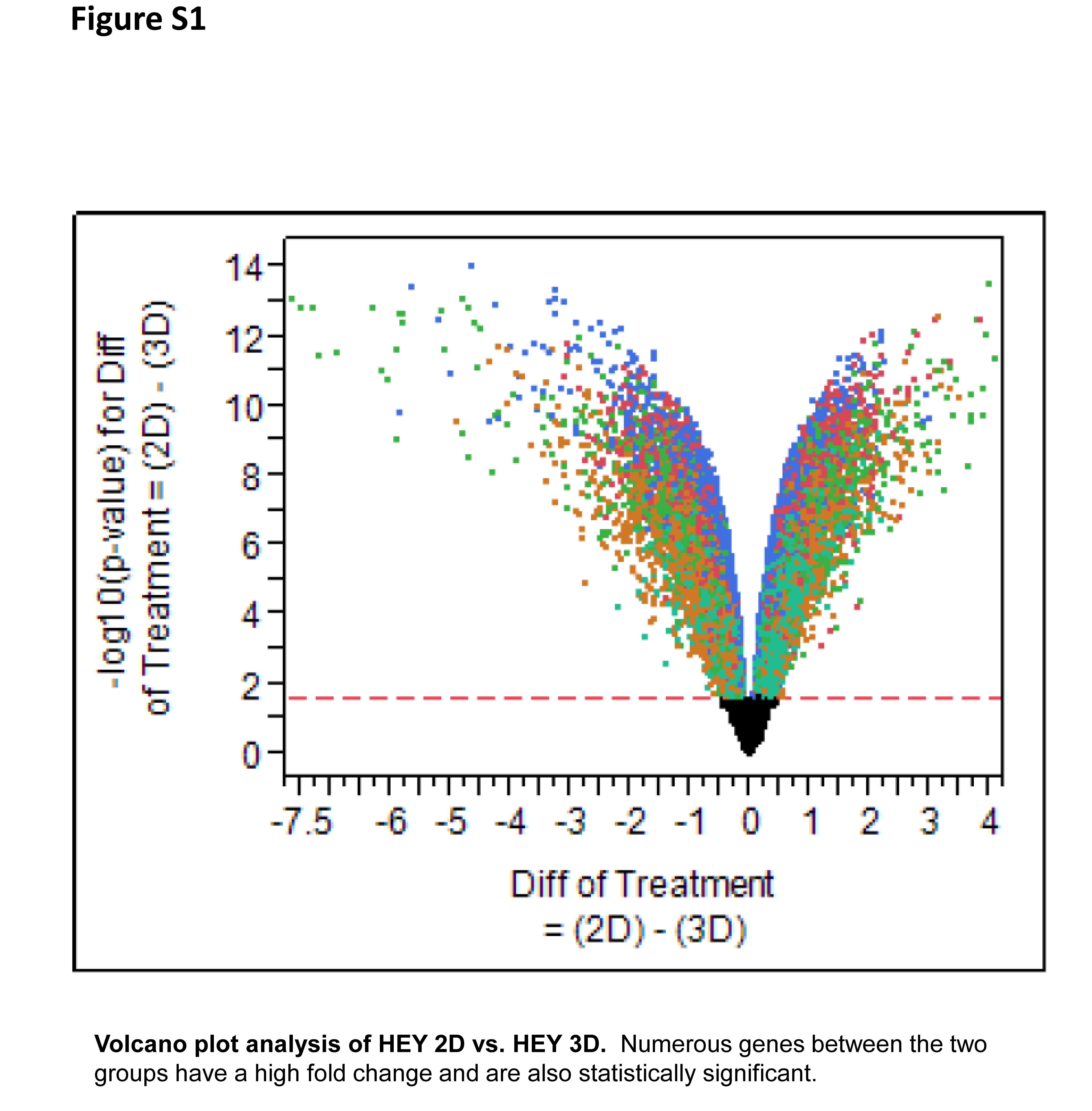

Supplement: S1 Fig — (TIF) [file pone.0182930.s001.tif]
