# Supplementary material for: Spheroid growth in ovarian cancer alters transcriptome responses for stress pathways and epigenetic responses
Source: PLoS One. 2017 Aug 9;12(8):e0182930. doi: 10.1371/journal.pone.0182930 (PMC5549971; doi:10.1371/journal.pone.0182930)
Supplement: S1 Table — (DOC) [file pone.0182930.s002.doc]

**Table S1 Primers for qPCR**

| Gene Name | Sequence |
| --- | --- |
| AHNAK2 | F: 5' - GAAAATCCCAGAGCCCCACA - 3' |
|  | R: 5' - GTGCCCTCCTGAGTCCTAGA - 3' |
| AKR1C1 | F: 5' - TGGCCATCCGAAGCAAGATT - 3' |
|  | R: 5' - GAGGATCATCTCCAGCTGCC - 3' |
| CCDC80 | F: 5' - CACGCAGAGTCCCAAGAAGT - 3' |
|  | R: 5' - CAAAATTGCACGCCTGACCA - 3' |
| HSPA1A | F: 5' - GAGGGCCATGACGAAAGACA - 3' |
|  | R: 5' - TCGCTGATCTTGCCCTTGAG - 3' |
| HSPH1 | F: 5' - AGGATCTCCCAAGCCTGGAT - 3' |
|  | R: 5' - TGGAGAAAGGAGCAGCATGG - 3' |
| PRSS35 | F: 5' - GCTGAAGCGTGCTCACAAAA - 3' |
|  | R: 5' - GTCGGACACACTGCAAAACC - 3' |
| RGS2 | F: 5' -TCTACTCCTGGGAAGCCCAA - 3' |
|  | R: 5' - GAGGACAGCTTTTGGGGTGA - 3' |
| RRAD | F: 5' - CCATGGGGGATGCCTATGTC - 3' |
|  | R: 5' - CGGCTGTTACGAGCTACGAT - 3' |
